# c-Jun N-terminal kinase activation has a prognostic implication and is negatively associated with FOXO1 activation in gastric cancer

**DOI:** 10.1186/s12876-016-0473-9

**Published:** 2016-06-06

**Authors:** Youngsun Choi, Jinju Park, Yiseul Choi, Young San Ko, Da-Ae Yu, Younghoon Kim, Jung-Soo Pyo, Bo Gun Jang, Min A. Kim, Woo Ho Kim, Byung Lan Lee

**Affiliations:** Department of Tumor Biology, Cancer Research Institute, Seoul National University College of Medicine, Seoul, 110-799 South Korea; Departments of Anatomy, Seoul National University College of Medicine, Seoul, 110-799 South Korea; Department of Pathology, Seoul National University College of Medicine, Seoul, 110-799 South Korea; Department of Pathology, Kangbuk Samsung Hospital, Sungkyunkwan University School of Medicine, Seoul, 110-746 South Korea; Department of Pathology, Jeju National University Hospital, Jeju, 690-767 South Korea; Ischemic/Hypoxic Disease Institute Medical Research Center, Seoul National University College of Medicine, Seoul, 110-799 South Korea

**Keywords:** JNK, Gastric cancer, Clinical significance, Proliferation, FOXO1

## Abstract

**Background:**

Since the biological function of c-Jun N-terminal kinase (JNK) in gastric cancer remains unclear, we investigated the clinical significance of JNK activation and its association with FOXO1 activation.

**Methods:**

Immunohistochemical tissue array analysis of 483 human gastric cancer specimens was performed, and the results of the immunostaining were quantified. The correlation between JNK activation (nuclear staining for pJNK) and clinicopathological features, the proliferation index, prognosis or FOXO1 inactivation (cytoplasmic staining for pFOXO1) was analyzed. The SNU-638 gastric cancer cell line was used for in vitro analysis.

**Results:**

Nuclear staining of pJNK was found in 38 % of the gastric carcinomas and was higher in the early stages of pTNM (*P* < 0.001). pJNK staining negatively correlated with lymphatic invasion (*P* = 0.034) and positively correlated with intestinal type by Lauren’s classification (*P* = 0.037), Ki-67-labeling index (*P* < 0.001), cyclin D1 (*P* = 0.045), cyclin E (*P* < 0.001) and pFOXO1 (*P* < 0.001). JNK activation correlated with a longer patients survival (*P* =0.008) and patients with a JNK-active and FOXO1-inactive tumor had a higher survival rate than the remainder of the population (*P* = 0.004). In vitro analysis showed that JNK inhibition by SP600125 in SNU-638 cells decreased cyclin D1 protein expression and increased FOXO1 activation. Further, JNK inhibition markedly suppressed colony formation, which was partially restored by FOXO1 shRNA expression.

**Conclusions:**

Our results indicate that JNK activation may serve as a valuable prognostic factor in gastric cancer, and that it is implicated in gastric tumorigenesis, at least in part, through FOXO1 inhibition.

## Background

Gastric cancer has been reported to be the fourth most common cancer and the second leading cause of cancer-related death worldwide [1]. More than 930,000 new cases are diagnosed and 700,000 deaths are attributed to gastric cancer annually [2]. Although gastric cancer develops through the accumulation of genetic alterations, such as oncogene (e.g. HER2, c-Met) activation and tumor suppressor gene (e.g. PTEN, p53) loss [3–6], the underlying molecular mechanisms of gastric carcinogenesis are largely unknown. Although anti-HER2 drugs such as trastuzumab and lapatinib are being used as molecularly targeted agents for gastric cancer patients with HER2 overexpression and patients responded clinically, acquired resistance to these anti-HER2 drugs has been observed in a subset of gastric cancer patients following chronic exposure [7]. Thus, better understanding of the molecular pathways involved in gastric cancer promotion will be helpful to improve gastric cancer therapies.

c-Jun N-terminal kinase (JNK) is a mitogen-activated protein kinase (MAPK), which regulates a wide range of cellular functions through both transcription-dependent and transcription-independent mechanisms [8]. In recent years, JNK has increasingly been recognized as an attractive molecular target for cancer treatment because of its broad roles in the regulation of cancer-associated cellular processes, including cell proliferation, differentiation and survival [8]. Since JNK acts as either a tumor promoter in breast cancer [9] and prostate cancer [10] or a tumor suppressor in skin cancer [11] and ovarian cancer [12], it is important to fully understand the role of JNK and the underlying molecular mechanisms in each tumor in order to validate the therapeutic potential of JNK.

With respect to gastric cancer, the biological significance of JNK has been controversial. Several in vitro studies have shown that JNK activation decreases gastric cancer cell survival [13–18]. In contrast, opposite finding have also been reported. JNK activation suppressed the apoptosis of gastric cancer cells in one study [19]. In addition, Shibata et al. [20] reported that JNK increased the development of gastric tumor in mice. Shibata et al. [20] also observed JNK activation in 40 % of 40 surgically resected human gastric carcinoma specimens. Thus, the role of JNK in gastric cancer remains elusive.

The subclass O of forkhead transcription factors (FOXO) consists of four members, FOXO1, FOXO3, FOXO4, and FOXO6. FOXO transcriptional activity is regulated by a complex array of post-translational modifications, including phosphorylation, acetylation, ubiquitination, and binding protein partners [21]. The two main post-translational modifications regulating FOXO phosphorylation are mediated by the AKT and JNK pathways [21]. Since FOXO proteins regulate diverse cellular functions, their dysregulations are considered to be potential targets of cancer therapy [22]. In gastric cancer, FOXO1 inactivation occurs and increases gastric cancer cell proliferation [23, 24] and angiogenesis [25]. Thus, restoration of FOXO1 activity may be a useful tool to suppress gastric tumor promotion.

JNK regulation of FOXO proteins is highly conserved across species [26], and a positive regulation of FOXOs by JNK has been shown in various human cancer cells, including colon cancer cells [27, 28], thyroid cancer cells [29], melanoma cells [30], and lung cancer cells [22, 31]. However, there has not been any study of that association in gastric cancer.

The present study was performed to evaluate the biological significance of JNK alone or in combination with FOXO1 in human gastric cancer regarding patient survival rate and tumor growth. Here, we evaluated the immunostaining for the active form of JNK phosphorylated at Thr183 and Tyr185 (pJNK) in 483 surgically resected human gastric carcinoma specimens and assessed its clinical significance. In cell culture experiments, SNU-638 gastric cancer cells were treated with a specific JNK inhibitor SP600125 to determine the direct role of JNK in the gastric cancer cell growth. In addition, the association between JNK and FOXO1 was examined in human gastric cancer specimens and gastric cancer cells.

## Methods

### Patients and tissue samples

Four hundred eighty-three surgically resected gastric carcinoma cases examined at the Department of Pathology, Seoul National University College of Medicine from 2 January to 29 December 2006 were analyzed. Gender, age, tumor size, tumor location, World Health Organization (WHO), Lauren’s classification, pathological tumor-node-metastasis (pTNM) stage and lymphatic invasion were evaluated by reviewing medical charts and pathological records [1], and examining the glass slides from each case. These information were summarized in Table 1. No patient received preoperative chemotherapy or radiotherapy. Patients’ clinical outcomes were followed from the date of surgery to either the date of death or 22 July 2013, resulting in the follow-up period ranged from 1 to 92 months. This protocol was reviewed and approved by the Institutional Review Board of Seoul National University (IRB No. 1309-087-522).

**Table 1:** Clinicopathological profiles of 483 gastric cancer patients

Clinicopathological features		Total cases (*n* = 483)
Gender	Male	337
	Female	146
Age (years)	Mean ± SD	58.3 ± 12.2
Tumor size (cm)	Mean ± SD	4.9 ± 3.1
Tumor location	Low	95
	Middle	165
	Upper	222
	Whole	0
	Remnant	1
WHO	Papillary	11
	WD	65
	MD	157
	PD	182
	Mucinous	17
	SRC	47
	Other	4
Laurens’ classification	Intestinal	236
	Diffuse	193
	Mixed	54
Tumor stage	I	271
(TNM stage according	II	91
to AJCC system)	III	62
	VI	59
Lymphatic invasion	Absent	273
	Present	210
Follow-up (months)	Range (Mean)	1-92 (69)

*SD* standard deviation, *WHO* World Health Organization

### Tissue array methods

Nine paraffin array blocks were prepared by Superbiochips Laboratories (Seoul, Korea), as previously described [32]. Briefly, core tissue biopsies (2 mm in diameter) were taken from individual paraffin-embedded gastric tumors (donor blocks) and arranged in a new recipient paraffin block (tissue array block) using a trephine apparatus. Each tissue array block was able to contain up to 60 cases, allowing nine array blocks to contain 483 cases. Each block contained an internal control consisting of non-neoplastic gastric mucosa from body, antrum and intestinal metaplasia. The staining results of the different intra-tumoral areas of gastric carcinomas in these tissue array blocks showed an excellent agreement [33] as described in the discussion section. A core was chosen from each case for analysis. We defined an adequate case as a tumor occupying more than 10 % of the core area.

### Immunohistochemistry

Immunohistochemistry was performed using the streptavidin-peroxidase technique. Paraffin-embedded sections of 4 μm thicknesses were cut from each tissue array block and mounted on glass slides. Then, the tissue array slides were deparaffinized at 59 °C for 1 h followed by xylene treatment. After hydration in an ethanol series, antigen retrieval was performed by microwave method: slides immersed in 0.01 M citrate buffer (pH 6.0) were placed in microwave oven and microwaved for 15 min (700 W, medium, low, each for 5 min). After cooling in cold PBS, endogenous peroxidase activity was quenched by incubating the slides in 3 % hydrogen peroxide for 10 min. Nonspecific binding was blocked by treating sections with 5 % normal goat serum or 2 % normal horse serum (Vectastain ABC kit; Vector Laboratories, Burlingame, CA, USA) for 1 h. Sections were incubated with primary antibodies against phospho-JNK^Thr183/Tyr185^ (1:50; rabbit polyclonal; Cell Signaling Technology, Beverly, MA, USA), phospho-FOXO1^Ser256^ (1:50; rabbit polyclonal; Cell Signaling Technology), Ki-67 (1:50; mouse monoclonal; DAKO, Glostrup, Denmark), cyclin D1 (1:500; rabbit polyclonal; Santa Cruz Biotechnology, Santa Cruz, CA, USA) or cyclin E (1:100; mouse monoclonal; Santa Cruz Biotechnology) at 4 °C overnight. After washing, sections were reacted with a secondary antibody, which is biotinylated goat anti-rabbit IgG (1:200, Vector Laboratories) or biotinylated horse anti-mouse IgG (1:100, Vector Laboratories) for 2 h at room temperature. Immunoreactions were visualized by incubation for 1 h at room temperature with streptavidin-horseradish peroxidase conjugate (Vector Laboratories), followed by a reaction with 0.025 % (w/v) 3,3-diaminobenzidine tetrahydrochloride (DAB) for 3 min and counterstaining with Meyer’s hematoxylin. Throughout the above analyses, controls were prepared by omitting the primary antibody. The expressions of proteins were assessed in a blinded fashion by two investigators (Y Kim and JS Pyo). For statistical analysis of immunostaining for proteins other than Ki-67, cases showing nuclear (pJNK, cyclin D1 and cyclin E) or cytoplasmic (pFOXO1) staining in 10 % or more of the tumor cells were considered to indicate positive staining. For Ki-67 staining, we evaluated 300 cells and counted the cells with nuclear staining for each specimen. The proliferation index was defined as follows: proliferation index (%) = 100 X Ki-67-positive cells/total cells.

### Cell culture

A human gastric cancer cell line SNU-638 was obtained from the Korean Cell Line Bank (Seoul, Korea). Cells were cultured in RPMI1640 (Life Technologies, Grand Island, NY, USA) supplemented with 10 % fetal bovine serum (FBS), 2 mg/mL sodium bicarbonate, 100 U/mL penicillin, and 100 μg/mL streptomycin (Life Technologies) at 37 °C in a humidified 95 % air and 5 % CO_2_ atmosphere.

### Treatment with a JNK inhibitor

To inhibit endogenous JNK activity, SNU-638 cells were treated with the indicated concentrations (5, 10, 20, and 30 μM) of a JNK inhibitor, SP600125 (Cell Signaling Technology), dissolved in dimethylsulfoxide (DMSO).

### Western blotting

Cell lysates were prepared in 100–200 μl of 1 x sodium dodecyl sulfate (SDS) lysis buffer [125 mM Tris–HCl (pH 6.8), 4 % SDS, 0.004 % bromophenol blue, and 20 % glycerol]. Protein contents were measured using BCA Protein Assay Reagent (Pierce, Rockford, IL, USA). Equal amounts of proteins were loaded onto a 10 % discontinuous SDS/polyacrylamide gel and electrophoretically transferred to PVDF membranes (Millipore Corporation, Billerica, MA, USA) blocked with 5 % nonfat dry milk in phosphate-buffered saline (PBS)-Tween 20 (0.1 %, v/v) for 1 h. The membranes were then incubated at 4 °C overnight with or without 2 h incubation at room temperature with one of the following primary antibodies: anti-pJNK (1:1000, Cell Signaling Technology), anti-total JNK (1:1000, Cell Signaling Technology), anti-cyclin D1 (1:1000, Santa Cruz Biotechnology), anti-pFOXO1 (1:1000, Cell Signaling Technology), anti-total FOXO1 (1:1000, Cell Signaling Technology), and anti-β-actin (1:1000, Santa Cruz Biotechnology). Horse-radish peroxidase-conjugated anti-rabbit IgG (1:2000; Santa Cruz Biotechnology) or anti-mouse IgG (1:2500; Santa Cruz Biotechnology) was used as a secondary antibody. Enhanced chemiluminescence was used to detect the immunoreactive proteins. Equal protein loading was confirmed by β-actin.

### Lentivirus-mediated short hairpin RNA (shRNA) silencing of FOXO1

FOXO1 shRNA lentiviral particles and non-targeting shRNA control particles were purchased from Sigma (St Louis, MO, USA). The sequence of the shRNA targeting FOXO1 used in the present study is the following: 5’-CCGGGCCTGTTATCAATCT-GCTAAACTCGAGTTTAGCAGATTGATAACAGGCTTTTTG-3’. The non-targeting shRNA control particles contain four base-pair mismatches within the short hairpin sequence to any known human or mouse gene. The viral infection was performed by incubating SNU-638 gastric cancer cells in the culture medium containing lentiviral particles for 12 h in the presence of 5 μg/mL Polybrene (Santa Cruz Biotechnology). Pooled puromycin (2 μg/mL)-resistant cells were harvested and stored for further analysis.

### Luciferase reporter assay

Gastric cancer cells were seeded into 24-well plates at a density of 3 × 10^4^ cells/well and were transiently co-transfected with 0.4 μg of forkhead responsive element (FHRE) -luciferase reporter plasmid (reporter construct in which a small region of the Fas ligand promoter containing the three FHREs, Addgene plasmid 1789) (Addgene Inco, Cambridge, MA, USA) and 0.4 μg of β-galactosidase vector, an internal control, using Lipofectamine Plus (Life Technologies). Twenty-four hours after transfection, assays for luciferase and β-galactosidase were carried out using a Dual-Luciferase Reporter Assay System (Promega, Madison, WI, USA). Luciferase activity was measured on an AutoLumat LB 9505c luminometer (Berthold Analytical Instruments, Nashua, Germany) and was normalized by β-galactosidase activity. Luciferase activity in control cells was arbitrarily set to 1.

### Immunofluorescence staining

Cells were cultured on 4-well chamber slide (3 × 10^4^ cells/chamber). After 24 h, cells were fixed with 4 % paraformaldehyde for 10 min and permeabilized with 0.5 % Triton X-100 for 5 min. After blocking with 5 % normal donkey serum for 5 min, cells were incubated overnight 4 °C with anti-pJNK (Cell Signaling Technology) and anti-FOXO1 (Cell Signaling Technology). Corresponding secondary antibody conjugated with Alexa Fluor-488 (green) or Alexa Fluor-555 (red) used for pJNK and FOXO1 respectively. For localization of nucleus, cells were counterstained with 4’,6-diamidino-2-phenylindole (DAPI) (Life Technologies) for 10 min. Immunofluorescence was detected under a fluorescence microscope (BX51; Olympus, Tokyo, Japan).

### Colony formation assay

Gastric cancer cells (2.5 × 10^3^ cells/well) were suspended in 0.3 % Bacto Agar (Sigma) in the RPMI1640 medium with 10 % FBS and overlaid onto a previously prepared 0.6 % Bacto Agar in 6-well culture plates (Thermo Scientific, Hudson, NH, USA). The agar plates were incubated with 1 mL of RPMI1640 medium, and medium was changed every 3 days. After 14 days of culture, surviving colonies were stained with 0.05 % crystal violet (Sigma) in 20 % methanol (Merck KGaA, Darmstadt, Germany). The number and size of colonies were determined using NIH Image Analysis software (version 1.46r; National Institutes of Health, Bethesda, MD, USA) as described previously [34]. For the inhibition of JNK activity, cells were cultured for 24 h in the media and treated with SP600125 (20 μM) for 14 days.

### Statistical analysis

For tissue array analysis, statistical analyses were conducted using SPSS version 19.0 statistical software program (IBM, SPSS, Chicago, Ill., USA). To determine the significance of correlation between JNK activation and the clinicopathological factors or the other proteins, either the *χ*^2^ test or the Fisher’s exact test (two sided) was performed. Survival curves were estimated using the Kaplan-Meier product-limit method and the significance of differences between the survival curves was determined using the log-rank test. To determine whether JNK activation is an independent prognostic variable, multivariate survival analysis was performed using the Cox proportional hazard model. The relationship between JNK activation and the cell proliferation index was analyzed using the two-tailed Student’s *t*-test. For luciferase reporter assay and colony formation assay, data were analyzed using GraphPad Prism software for Windows Vista (version 4, San Diego, CA, USA) and the two-tailed Student’s *t*-test was used to determine the significances of the results. *P* values of < 0.05 were considered statistically significant for all statistical analyses.

## Results

### Immunohistochemical features

**Fig. 1:**
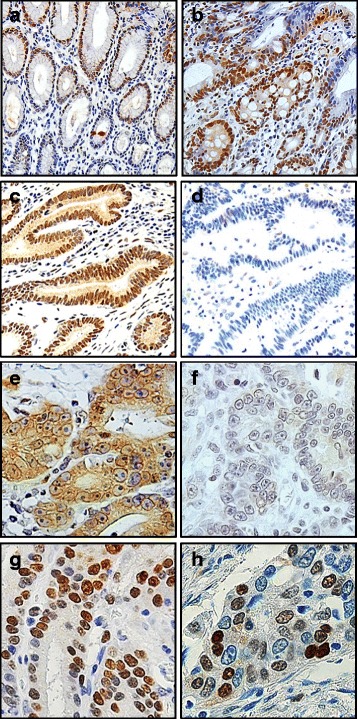
Representative immunohistochemical features of the gastric mucosa and cancer. Nuclear pJNK was expressed (**a**) in the proliferative zone of gastric mucosa and (**b**) in the areas of intestinal metaplasia. Gastric carcinomas displaying (**c**) positive or (**d**) negative expression of pJNK. Gastric carcinomas displaying (**e**) positive or (**f**) negative expression of pFOXO1. Gastric carcinoma cells showing nuclear expression of (**g**) Ki-67 or (**h**) cyclin D1. Original magnifications: ×100 (**a**-**d**) and × 400 (**e**-**h**)

Figure 1 shows the representative findings of immunohistochemical tissue array analysis of 483 consecutive gastric carcinoma tissues. JNK activation was determined by immunostaining using an antibody against the active form of JNK (pJNK). In non-neoplastic gastric mucosa, pJNK (immuno)staining was observed throughout the nucleus of the proliferative zone of gastric glands (Fig. 1a). Nuclear pJNK staining was also detected in the areas of intestinal metaplasia with or without cytoplasmic staining (Fig. 1b). Gastric tumor cells showed pJNK staining mainly in the nucleus and/or in the cytoplasm (Fig. 1c), and nuclear staining was the criterion for pJNK [35, 36].

For FOXO1, immunostaining was performed with an antibody against an inactivated form of FOXO1 phosphorylated at Ser 256 (pFOXO1). pFOXO1 staining was observed in the cytoplasm and, at times, in the nucleus of tumor cells (Fig. 1e). Tumor cells with cytoplasmic staining regardless of the nuclear staining were considered to exhibit FOXO1 inactivation [23]. For other proteins such as Ki-67 (Fig. 1g) and cyclin D1 (Fig. 1h), tumor cells with nuclear staining, regardless of cytoplasmic staining, were regarded as showing constitutive activation. For statistical analysis, immunohistochemical staining results were quantified as described in Methods.

### JNK activation is associated with the clinicopathological factors in gastric cancer

In order to investigate the clinical significance of JNK activation in gastric cancer, the correlations between nuclear pJNK staining and the clinicopathologic features in 483 gastric cancer cases were analyzed (Table 2). Nuclear pJNK staining was found in 38 % of gastric cancer cases and was more likely to be found in the intestinal type by Lauren’s classification (*P* = 0.037). In addition, pJNK staining was more prevalent in the early pTNM stages (*P* < 0.001). Eighty-two percent of pJNK-positive tumors were in pTNM stages I and II, while 18 % were in stages III and IV. Moreover, pJNK staining inversely correlated with lymphatic invasion (*P* = 0.034). No association was found between JNK activation and age or gender.

**Table 2:** Correlation between clinicopathological characteristics and pJNK expression in 483 gastric cancers

	pJNK		*P*
	Positive (%)	Negative (%)	
Total	182 (38)	301 (62)	
Age (years)			
0–39	13 (36)	23 (64)	0.432
40–65	103 (36)	186 (64)	
66–88	66 (42)	92 (58)	
Gender			
Male	134 (40)	203 (60)	0.091
Female	48 (33)	98 (67)	
Lauren’s classification			
Intestinal	104 (44)	132 (56)	0.037*
Diffuse	62 (32)	131 (68)	
Mixed	16 (30)	38 (70)	
pTNM stage			
I	127 (47)	144 (53)	<0.001*
II	22 (24)	69 (76)	
III	21 (34)	41 (66)	
IV	12 (20)	47 (80)	
Lymphatic invasion			
Absent	113 (41)	160 (59)	0.034*
Present	69 (33)	141 (67)	

*pJNK* phosphorylated c-Jun N-terminal kinase, *TNM* tumor-node metastasis

*Considered to be statistically significant (<0.05)

### Patients with a pJNK-positive tumor showed a higher survival rate

**Fig. 2:**
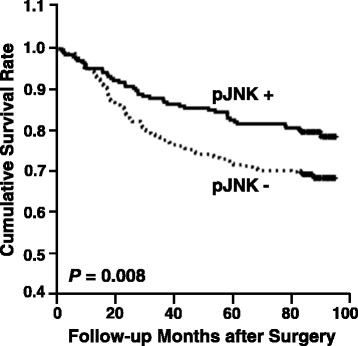
Kaplan-Meier survival curves for patient survival. Patients with a pJNK-positive tumor showed a more favorable prognosis than pJNK-negative carcinoma patients (*P* = 0.008)

To determine whether JNK activation is a significant prognostic factor for the survival of patients with gastric carcinoma, we used a log-rank test with Kaplan-Meier estimates. Of the 483 patients analyzed, those with nuclear pJNK staining (38 %) had a significantly higher survival rate than those with only cytoplasmic pJNK staining or negative staining (*P* = 0.008) (Fig. 2). Thus, JNK activation is a valuable biomarker of prognosis. However, multivariate Cox regression analysis including the pTNM stage revealed that pJNK staining was not an independent prognostic factor.

### JNK activation is positively associated with the tumor cell proliferation and growth

**Fig. 3:**
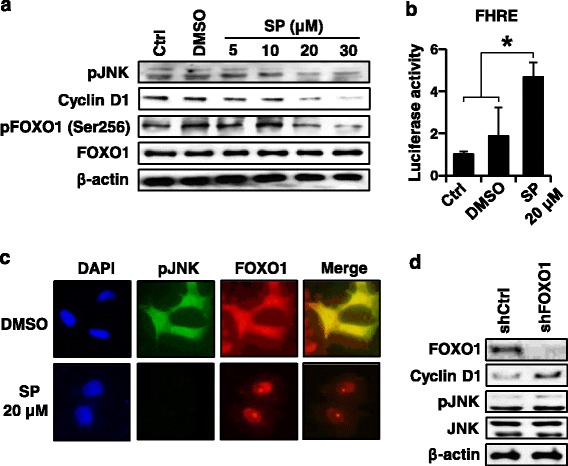
The effect of pharmacological inhibition of JNK on FOXO1 activation in cultured gastric cancer cells. (**a**-**c**). SNU-638 cells were cultured without treatment (Ctrl) or treated with DMSO vehicle control (DMSO) or SP600125 (SP) for 24 h. **a** Cells were treated with various concentrations of SP. Protein expressions were analyzed by Western blotting using specific antibodies against pJNK^Thr183/Tyr185^ (pJNK), cyclin D1, pFOXO1^Ser256^, total FOXO1, and β-actin. **b** Cells were cultured in the presence or absence of SP (20 μM) and FOXO1 transcriptional activity was determined by luciferase reporter assay, which was normalized by β-galatosidase activity. Luciferase activity in Ctrl was arbitrarily set to 1 and the activities of other cells were adjusted accordingly. Each bar represents the mean ± standard deviation. * *P* < 0.05 compared with untreated control groups (Ctrl and DMSO). **c** The effect of JNK inhibition on the subcellular localization of FOXO1 was observed by double immunofluorescence staining for pJNK (green) and FOXO1 (red). 4’,6-diamidino-2-phenylindole (DAPI) staining was performed for nuclear localization (blue). **d** Cells were transfected with non-targeting shRNA (shCtrl) or FOXO1 shRNA (shFOXO1). The effects of FOXO1 silencing on the expressions of total FOXO1, cyclin D1, pJNK, and total JNK were evaluated by Western blotting

**Fig. 4:**
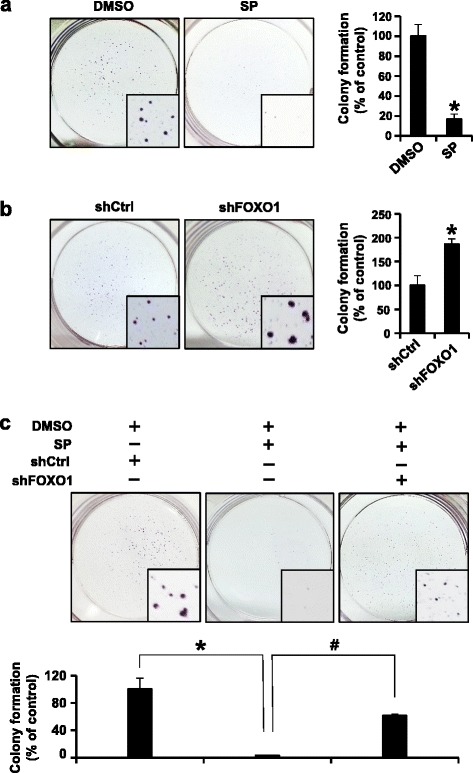
Role of JNK and FOXO1 in gastric tumor cell growth. SNU-638 cells were (**a** and **c**) cultured with or without 20 μM SP600125 (SP) for 14 days and/or (**b** and **c**) transfected with non-targeting shRNA (shCtrl) or FOXO1 shRNA (shFOXO1). Cell growth ability in vitro was evaluated by colony formation assay. Representative images of colonies are shown and the bar graphs represent the relative colony formation efficiencies. Percentage colony formations for control cells were arbitrarily set to 100 % and percentages for others were adjusted accordingly. **a** SP-treated cells showed a marked reduction in colony formation compared to DMSO control (* *P* = 0.013). **b** shFOXO1 transfection induced a significant enhancement in colony formation compared to shCtrl transfection (* *P* = 0.021). **c** SP treatment induced a marked suppression in colony formation compared to DMSO + shCtrl (* *P* = 0.01), and the combination with shFOXO1 transfection partially restored the colony formation compared to SP treatment (^#^ *P* < 0.001). Each bar represents the mean ± standard deviation

Tumor cell proliferation was evaluated by the immunohistochemical stainings for nuclear Ki-67 and cell cycle regulators, including cyclin D1 and cyclin E. Table 3 shows the results of the immunohistochemistry for Ki-67 performed on tissue array slides. pJNK staining positively correlated with the proliferation index (evaluated by Ki-67 labeling) (*P* < 0.001). Moreover, Table 4 shows positive correlations between pJNK and cyclin D1 (*P* = 0.045) or cyclin E (*P* < 0.001). Consistently, Western blotting of cultured SNU-638 cells showed that JNK inhibition by SP600125 treatment suppressed the protein expressions of pJNK and cyclin D1 in a dose-dependent manner (Fig. 3a). Taken together, our data from both human specimens and in vitro experiments suggested that JNK may promote gastric cancer cell proliferation through enhancement of cell cycle progression. Further, SP600125 treatment of gastric cancer cells suppressed colony formation of SNU-638 cells (Fig. 4a).

**Table 3:** The expression of pJNK in relation to the proliferation index

	pJNK		*P*
	Positive (*n* = 182) Mean ± SD (%)	Negative (*n* = 301) Mean ± SD (%)	
Ki-67 (%)	86.78 ± 51.34	54.65 ± 46.53	<0.001*

*SD* standard deviation

*Considered to be statistically significant (<0.05)

**Table 4:** Correlation between the expression of pJNK and protumorigenic molecules in human gastric cancer

	pJNK		*P*
	Positive (%)	Negative (%)	
Cyclin D1			
Positive	79 (42)	107 (58)	0.045*
Negative	97 (34)	186 (66)	
Cyclin E			
Positive	41 (58)	30 (42)	<0.001*
Negative	139 (34)	266 (66)	

*Considered to be statistically significant (<0.05)

### Association between JNK and FOXO1 in gastric cancer specimens

To obtain a better understanding of the mechanism involved in the JNK-induced gastric tumor cell proliferation, we investigated the relationship between JNK and FOXO1. Immunohistochemical tissue array analysis showed that JNK activation was positively associated with FOXO1 inactivation in gastric cancer specimens, which indicates an inverse relationship between the activations of JNK and FOXO1 (*P* < 0.001) (Table 5). Consistently, FOXO1 inactivation, like JNK activation, was positively associated with the nuclear stainings for cyclin D1 (*P* = 0.003) and cyclin E (*P* < 0.001). In addition, FOXO1 staining in gastric cancer was not an independent prognostic factor.

**Table 5:** Correlation between pFOXO1 expression and pJNK expression and protumorigenic molecules in gastric cancer

	pFOXO1		*P*
	Positive (%)	Negative (%)	
pJNK			
Positive	169 (93)	13 (7)	<0.001*
Negative	206 (68)	95 (32)	
Cyclin D1			
Positive	157 (84)	29 (16)	0.003*
Negative	208 (73)	75 (27)	
Cyclin E			
Positive	68 (96)	3 (4)	<0.001*
Negative	301 (74)	104 (26)	

*Considered to be statistically significant (<0.05)

### Pharmacological inhibition of JNK results in FOXO1 activation in gastric cancer cells

To further confirm the relationship between JNK and FOXO1, in vitro analysis was performed. First, SNU-638 gastric cancer cells were treated with a JNK inhibitor, SP600125. Western blotting (Fig. 3a) showed that SP600125 treatment of gastric cancer cells with a various concentrations resulted in a substantial decrease in the protein expression of the inactive form of FOXO1 (pFOXO1) in a dose dependent manner, whereas the protein expression of total FOXO1 was not changed. Consistently, luciferase reporter assay (Fig. 3b) showed that treatment of SNU-638 cells with SP600125 (20 μM) increased FOXO1 transcriptional activity compared to those without treatment (Ctrl and DMSO) with a statistical significance (*P* = 0.003 and *P* = 0.025, respectively).

Next, immunofluorescence stainings for pJNK and FOXO1 were performed to determine the effect of JNK inhibition on the subcellular localization of FOXO1 in SNU-638 cells (Fig. 3c). In the untreated DMSO control cells (upper row), immunofluorescence for pJNK (green) was shown both in the nucleus and the cytoplasm. After pharmacological inhibition of JNK with 20 μM SP600125 (lower row), gastric cancer cells showed negligible immunofluorescence for pJNK. On the other hand, immunofluorescence for FOXO1 was distributed both in the nucleus and the cytoplasm in control cells, whereas it was only accumulated in the nucleus of the SP600125-treated cells. Thus, it seems that JNK functions in the nucleus and that the translocation of FOXO1 from the nucleus to the cytoplasm was blocked by JNK inhibition. These findings are incompatible to previous reports in human cancer cells, which showed that JNK activation induced the nuclear translocation of FOXO1 proteins followed by an increase in FOXO transcriptional activity [22, 27–31].

In order to determine whether there is crosstalk between JNK and FOXO1, FOXO1 expression was silenced by transfection of FOXO1 shRNA into SNU-638 cells. Western blotting showed that FOXO1 silencing did not change protein expressions of pJNK and total JNK, whereas it increased cyclin D1 protein expression compared to control shRNA transfectants (Fig. 3d). Taken together, our data indicate that JNK acts as an inhibitory upstream molecule of FOXO1 in the FOXO1 pathway in gastric cancer cells, and that crosstalk between these molecules does not exist.

### JNK induces gastric cancer cell growth through inhibition of FOXO1

The above results indicate that JNK activation in gastric cancer cells is associated with tumor cell proliferation and inhibition of FOXO1 activation. In order to determine the biological significance of the association between JNK and FOXO1 regarding tumorigenesis, colony formation assay was performed using SNU-638 cells. We found that SP600125 treatment markedly decreased colony formation (*P* = 0.013) (Fig. 4a), whereas FOXO1 silencing induced a significant increase in colony formation (*P* = 0.021) (Fig. 4b). In order to investigate whether JNK-induced colony formation is mediated by FOXO1 inactivation in gastric cancer cells, we used a combination of JNK inhibition and FOXO1 knockdown. In Fig. 4c, JNK inhibition markedly decreased colony formation (*P* = 0.01), and FOXO1 shRNA transfection combined with JNK inhibition substantially increased colony formation compared to JNK inhibition alone (*P* < 0.001). These results indicate that JNK activation, which induces gastric cancer cell growth, is mediated by FOXO1 inactivation. Thus, JNK/FOXO1 pathway seems to contribute to tumor growth of gastric cancer.

### Combined status of pJNK-negative and pFOXO1-negative in relation to prognosis

**Fig. 5:**
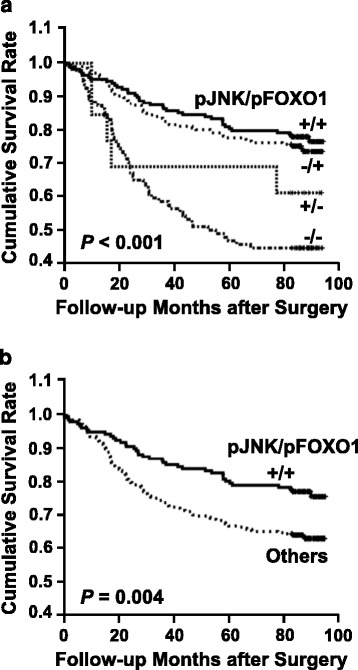
Kaplan-Meier survival curves for patient survival. **a** Patients with pJNK negative and pFOXO1-negative immunoreactivity showed the worst outcome (*P* < 0.001). **b** Patients with a pJNK-positive and pFOXO1-positive combination showed better outcome than the remainder of the population (*P* = 0.004)

We examined whether the combined status of pJNK and pFOXO1 is correlated with gastric cancer patient survival. Kaplan-Meier estimate and the log-rank test showed that patients with a pJNK-negative (JNK inactive) and pFOXO1-negative (FOXO1 active) tumor had significantly worse outcome than those with other combinations (*P* < 0.001) (Fig. 5a). Moreover, patients with a pJNK-positive (JNK active) and pFOXO1-postive (FOXO1 inactive) tumor had a higher survival rate than the remainder of the population (*P* = 0.004) (Fig. 5b).

**Fig. 6:**
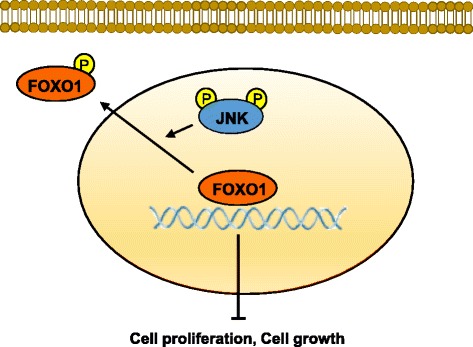
Schematic diagram showing the potential mechanism of cancer cell proliferation and growth regulated by the JNK/FOXO1 pathway in gastric cancer. As depicted, JNK activation induces cancer cell proliferation and growth by suppressing the transcriptional activity of FOXO1. When JNK is activated by phosphorylation, FOXO1 is phosphorylated, leading to its nuclear export and inactivation

## Discussion

Although accumulating evidence supports that JNK activation is involved in cancer development and progression [37, 38], the biological significance of JNK in gastric cancer remains unclear. The present study showed that constitutive activation of JNK was associated with specific clinicopathological factors, including pTNM stages, lymphatic invasion, and a better prognosis. We believe that this is the first report regarding the clinical implications of JNK in human gastric cancer. Furthermore, we found that JNK negatively regulates FOXO1 activation in gastric cancer cells. This finding contrasts with the results of the previous studies [22, 27–31], which showed JNK-induced activation of FOXO proteins in human cancer cells.

In the present study, JNK activation (evaluated by pJNK staining) was mainly observed in the proliferative zone of the gastric gland and in the areas showing intestinal metaplasia, which is known to be a predictor of gastric neoplasia [39], in the non-neoplastic gastric mucosa. Since the intestinal metaplasia shows a higher proliferation index than the normal gastric mucosa [40], this staining pattern suggested a positive association between JNK activation and cell proliferation. Moreover, JNK activation in gastric cancer was positively correlated with the proliferation index (evaluated by Ki-67 labeling) and cell cycle-regulatory molecules such as cyclin D1 (*P* = 0.045) and cyclin E (*P* < 0.001), which are present more frequently in early-stage gastric carcinomas [41]. In cell culture, treatment of gastric cancer cells with a JNK inhibitor, SP600125, decreased cyclin D1 protein expression and colony formation. Taken together, these findings indicate growth-promoting activity of JNK in gastric cancer cells, which is a very important process in the gastric cancer promotion.

Previously, Shibata et al. [20] reported that JNK activation was found in 40 % of gastric cancer cases and that there was no association between JNK activation and Lauren’s classification. However, our results showed that JNK activation was found in 38 % of 483 gastric carcinoma cases and was more likely to be found in the intestinal type as determined by Lauren’s classification (*P* = 0.037). We speculate that these discrepancies between the results of Shibata et al. and ours may, at least in part, come from differences in the numbers of tumor cases analyzed (*n* = 40 versus *n* = 483 in the present study). Moreover, we found that JNK activation in gastric cancer was more prominent in early-stage pTNM tumors than in late-stage pTNM tumors (*P* < 0.001), and was negatively associated with lymphatic invasion (*P* = 0.034). Thus, it seems that JNK activation, at least in part, is required for cell proliferation and growth in early stage gastric carcinomas, which do not usually show lymphatic invasion.

Although previous studies suggested that FOXO1 inactivation may contribute to the development of gastric cancer [23–25], information on the molecular mechanisms underlying FOXO1 activation in gastric cancer is limited. Regarding the association between FOXO1 and JNK, there has been only one study by Ju et al. [22], which showed that JNK induced FOXO1 activation in lung cancer cells. In the present study, immunohistochemical tissue array analysis of gastric cancer specimens showed that JNK activation was positively correlated with FOXO1 inactivation. This relationship was further confirmed by in vitro analyses using a JNK inhibitor, SP600126. JNK inhibition in SNU-638 cells increased FOXO1 activation and suppressed the nuclear export of FOXO1, which suggested that FOXO1 is a nuclear substrate of JNK in gastric cancer cells. However, crosstalk between JNK and FOXO1 was not observed, because FOXO1 silencing did not affect the protein expression and activity of JNK. Thus, it seems that JNK is an upstream regulatory molecule of FOXO1, which increases the nuclear export and subsequent inactivation of FOXO1 (Fig. 6). These findings contrast with a previous report by Ju et al. [22]. Thus, we speculate that the relationship between these two molecules could be cell type-specific.

Additionally, we found that colony formation of gastric cancer cells was decreased by pharmacological inhibition of JNK, whereas it was increased by FOXO1 shRNA transfection. Further, the combination of JNK inhibition and FOXO1 silencing partially restored the colony forming capability of gastric cancer cells compared to JNK inhibition alone. These results indicate that JNK activation, at least in part, induces gastric cancer cell growth through the inhibition of FOXO1. Since Altan et al. [42] reported that FOXO1 downregulation in a subset of gastric cancer cell lines inhibited 5-fluorouracil sensitivity, a JNK inhibitor may be used to restore the chemosensitivity to 5-fluorouracil through FOXO1 activation in the treatment of those gastric cancer patients.

Our results in the present study showed that JNK activation in gastric cancer specimens was positively correlated with early pTNM stages and with a better outcome. Similarly, FOXO1 inactivation was previously shown to be an early event and was positively correlated with better prognosis in gastric cancer [23]. Since gastric cancer specimens showed a positive correlation between JNK activation and FOXO1 inactivation in the present study, the combined status of JNK activation and FOXO1 inactivation was assessed in relation to survival. We found that patients with a tumor showing JNK activation and FOXO1 inactivation had a higher survival rate than those with other combinations (*P* = 0.004).

Tissue array method was used in the present study to evaluate the clinical significance of JNK activation and its association with FOXO1 inactivation. The potential limitations of tissue array method are mainly associated with the acquisition of information from only a tiny area in each tumor. In order to address the influence of tumor heterogeneity and to evaluate the ability of the tissue array method to yield information on the prognostic value of biomarkers, multiple replicate tissue array blocks had been constructed by several researchers [33, 43, 44]. In all of these studies, the data from each replica array showed an excellent agreement, i.e. they were almost identical within the statistical level of significance, and the prognostic associations of the markers were always as good as or better, when measured from the tissue array slides, than the analysis of individual large sections. Thus, it has been suggested that the effects of intra-tumoral heterogeneity were averaged out in such large scale analysis. Indeed, the tissue array method is used for population-level research to find molecular targets with therapeutic significance, but not for making clinical diagnoses of individual cases. In addition, core sampling from different tumor blocks from the same patient, perhaps including metastatic sites, are being used to compensate these limitations [45].

## Conclusions

Our results indicate that JNK activation, alone or along with FOXO1 inactivation, is a candidate prognostic marker for the early gastric carcinoma and positively associated with pro-proliferation genes and FOXO1 inactivation. Since JNK activation increased gastric cancer cell growth through inhibition of FOXO1, combination of targeting JNK/FOXO1 pathway may further reduce gastric cancer cell growth. In-vivo experiments using this association as a potential target for anti-cancer therapy are needed.

## Abbreviations

DMSO, dimethylsulfoxide; FBS, fetal bovine serum; FHRE, forkhead responsive element; FOXO, subclass O of forkhead transcription factors; JNK, c-Jun N-terminal kinase; MAPK, mitogen-activated protein kinase; PBS, phosphate-buffered saline; pFOXO1, inactive form of FOXO1 phosphorylated at Ser256; pJNK, active form of JNK phosphorylated at Thr183 and Tyr185; pTNM, pathological tumor-node-metastasis; SDS, sodium dodecyl sulfate; shRNA, short hairpin RNA
